# Temporal inverted internal limiting membrane flap technique versus conventional internal limiting membrane peeling technique for large macular holes: a retrospective comparative study

**DOI:** 10.1186/s12886-026-05228-3

**Published:** 2026-08-12

**Authors:** Sule Acar Duyan, Gulay Guler Canozer, Saban Gonul

**Affiliations:** 1 Department of Ophthalmology, Selçuk University Faculty of Medicine, Konya, Turkey; 2 Department of Ophthalmology, Konya City Hospital, Konya, Turkey

**Keywords:** Macular hole, Temporal inverted ILM flap, Conventional ILM peeling, Ellipsoid zone, External limiting membrane, Optical coherence tomography

## Abstract

**Background:**

Full-thickness macular holes (FTMHs) larger than 400 μm remain a surgical challenge with suboptimal closure rates when treated with conventional internal limiting membrane (ILM) peeling. The temporal inverted ILM flap technique has emerged as a promising alternative, yet direct comparisons-particularly regarding hole size specific outcomes and outer retinal microstructural recovery-remain scarce. This study aimed to compare the functional, anatomical, and morphological outcomes of these two surgical approaches.

**Methods:**

This retrospective study included 32 patients with large idiopathic FTMHs (> 400 μm) who underwent surgery between 2020 and 2024. Patients were divided into two groups: conventional ILM peeling (Group 1, *n* = 16) and temporal inverted ILM flap technique (Group 2, *n* = 16). All procedures were performed by a single experienced surgeon. Best-corrected visual acuity (BCVA), MH closure rate, closure configuration, diameter hole index (DHI), and outer retinal layer parameters including ellipsoid zone (EZ) and external limiting membrane (ELM) integrity were assessed using spectral-domain optical coherence tomography (SD-OCT) at baseline and 12 months postoperatively.

**Results:**

Baseline characteristics did not differ significantly between the groups (all *p* > 0.05). Both groups showed significant improvement in BCVA after surgery (both *p* < 0.05). At 12 months, postoperative BCVA was 0.71 ± 0.46 logMAR in Group 1 and 0.38 ± 0.32 logMAR in Group 2, with significantly better BCVA in Group 2 (*p* = 0.039). Primary closure was achieved in 75.0% (12/16) of eyes in Group 1 and 100% (16/16) in Group 2, although the overall difference was not statistically significant (*p* = 0.101). In an exploratory post hoc subgroup analysis of eyes with a minimum hole diameter ≥ 550 μm, primary closure occurred in 33.3% (2/6) of Group 1 eyes and 100% (8/8) of Group 2 eyes (*p* = 0.015); both groups achieved 100% closure in holes measuring 400–549 μm. Postoperative EZ defect size was 1028.62 ± 606.93 μm in Group 1 and 539.12 ± 618.09 μm in Group 2, with a significantly smaller defect size in Group 2 (*p* = 0.040).

**Conclusions:**

In this retrospective, non-randomized cohort, the temporal inverted ILM flap technique was associated with better postoperative BCVA and a smaller postoperative EZ defect size than conventional ILM peeling. Although an exploratory post hoc subgroup analysis suggested a higher primary closure rate in Group 2 among eyes with a minimum hole diameter ≥ 550 μm, the overall closure-rate difference was not statistically significant, and the subgroup analysis was based on very small sample sizes. Further prospective studies with larger sample sizes are needed to confirm these findings.

**Trial registration:**

Not applicable (retrospective study).

## Background

Full-thickness macular hole (FTMH) is characterized by a tissue defect extending from the internal limiting membrane (ILM) to the photoreceptor layer at the fovea [1, 2]. This condition frequently results in significant central vision loss, central scotoma, metamorphopsia, and other visual disturbances [3].

The International Vitreomacular Traction Study Group has established a size-based classification system for macular holes (MHs) using the minimum hole diameter: small (≤ 250 μm), medium (251–400 μm), and large (> 400 μm) [4].

Pars plana vitrectomy for MH repair was first described by Kelly and Wendel in 1991 [5]. The conventional ILM peeling technique has been reported to achieve closure rates of 80%–100% [6, 7]. Nevertheless, outcomes are closely linked to hole size, with minimum linear diameters exceeding 400–500 μm exhibiting lower closure rates [8, 9]. The temporal inverted ILM flap technique (IFT), originally described by Michalewska et al. [10, 11], has demonstrated higher closure rates in larger MHs compared with conventional ILM peeling.

Spectral-domain optical coherence tomography (SD-OCT) has enhanced postoperative assessment by providing detailed visualization of photoreceptor integrity, with restoration of the EZ and ELM closely associated with improved visual outcomes [12–14]. Only a limited number of studies have directly compared conventional ILM peeling and the temporal inverted ILM flap technique with respect to outer retinal microstructural recovery. The present study aimed to compare these two surgical approaches in the primary treatment of large idiopathic MHs.

## Methods

### Study design and participants

This retrospective comparative study included patients who underwent surgery for large idiopathic MHs (> 400 μm) at our institution between 2020 and 2024. Treatment allocation was not randomized and was not based on macular hole size, baseline visual acuity, lens status, axial length, symptom duration, or any other patient-specific characteristic. Rather, the choice of surgical technique was determined chronologically according to the surgical practice at our institution. Conventional ILM peeling was the routine approach during the earlier part of the study period, after which the temporal inverted ILM flap technique was adopted as the routine approach for large macular holes. All consecutive patients who met the eligibility criteria during the respective periods were included, and no additional case selection or matching was performed. Accordingly, patients were classified as those who underwent conventional ILM peeling (Group 1, *n* = 16) and those who underwent the temporal inverted ILM flap technique (Group 2, *n* = 16). The study was approved by the institutional clinical research ethics committee (Selçuk University Medical Faculty, 2024/502) and conducted in accordance with the principles of the 1964 Declaration of Helsinki. Written informed consent was obtained from all participants.

Inclusion criteria were: age ≥ 18 years, minimum MH diameter > 400 μm, and minimum postoperative follow-up of 12 months. Exclusion criteria included recurrent or traumatic MH; myopic MH, defined as myopic MH occurring in an eye with an axial length > 26.0 mm measured preoperatively by optical biometry (Lenstar LS 900; Haag-Streit Diagnostics, Köniz, Switzerland); coexisting retinal diseases other than MH; any ocular comorbidity potentially affecting visual outcomes; and prior retinal surgery.

### Clinical assessment

All patients underwent comprehensive ophthalmological examination at baseline and at 12 months postoperatively, including BCVA measurement, slit-lamp biomicroscopy, posterior segment evaluation with a 90-diopter non-contact lens, and SD-OCT (Heidelberg Engineering, Heidelberg, Germany). BCVA was measured using a Snellen chart and converted to the logarithm of the minimum angle of resolution (logMAR) for statistical analysis. Minimum and base MH diameters, diameter hole index (DHI; calculated as the minimum hole diameter divided by the base diameter), closure configuration, and preoperative and postoperative EZ and ELM defect sizes were assessed using SD-OCT. Postoperative EZ and ELM defect sizes and macular hole closure configuration were independently evaluated by two graders who were masked to the surgical technique. The graders assessed the images independently, and any disagreement was resolved by adjudication by a third independent grader who was also masked to treatment allocation. Symptom duration was defined as the interval between the patient-reported onset of central visual symptoms and the date of surgery, and was retrospectively extracted from clinical records for all patients.

### Surgical techniques

All procedures were performed by a single experienced surgeon using a standard 25-gauge pars plana vitrectomy system (Constellation Vision System, Alcon Laboratories, Fort Worth, TX, USA). Following core and cortical vitreous removal, the ILM was stained with membrane blue dye and any epiretinal membrane was peeled if present.

In Group 1, conventional ILM peeling was performed in a circular fashion approximately two disc diameters around the MH.

In Group 2, the temporal inverted ILM flap technique was performed as follows. After ILM staining, peeling was initiated approximately two disc diameters temporal to the MH. The ILM was carefully dissected in a temporal-to-nasal direction, creating a flap that remained attached at the temporal edge of the MH. The flap was then inverted and folded over the MH to cover it completely, with the inner surface of the ILM facing the hole. Fluid–air exchange was subsequently performed to stabilize the flap without the use of perfluorocarbon liquid or ophthalmic viscoelastic devices.

All eyes received 14% octafluoropropane (C3F8) gas tamponade at the conclusion of surgery. Patients were instructed to maintain a face-down (prone) position for 5 days postoperatively.

### Statistical analysis

Statistical analyses were performed using SPSS version 21.0 (SPSS Inc., Chicago, IL, USA). Continuous variables are expressed as mean ± standard deviation or median (interquartile range). Normality was assessed using the Shapiro-Wilk test, given the limited sample size. Between-group comparisons of normally distributed data used the independent samples t-test; non-normally distributed data were compared using the Mann-Whitney U test. Within-group preoperative and postoperative comparisons were performed using paired tests appropriate to the distribution of the data. Categorical variables were compared using the chi-square test or Fisher’s exact test. For the primary closure outcome, which contained a zero cell because no closure failure occurred in Group 2, a corrected odds ratio was calculated using the Haldane-Anscombe correction by adding 0.5 to each cell. A Wald-type 95% confidence interval was subsequently calculated on the log odds-ratio scale. The 95% confidence interval for the mean postoperative BCVA difference was obtained by bootstrap resampling (10,000 resamples), as this variable was not normally distributed. A multivariable linear regression model was constructed for postoperative BCVA, adjusting for surgical technique, age, lens status, DHI, baseline BCVA, and symptom duration. A corresponding logistic regression model for the primary closure outcome could not be reliably estimated owing to complete separation and the limited number of closure failures, and was therefore not interpreted. Given the limited sample size, the multivariable analysis was considered exploratory. The ≥ 550 μm subgroup cutoff used in the hole-size stratified analysis was determined post hoc, based on inspection of the observed distribution of minimum hole diameters, and was not prespecified; no receiver operating characteristic or other threshold-optimization analysis was performed to validate it. A p-value of < 0.05 was considered statistically significant.

### Use of artificial intelligence

Artificial intelligence (AI) language tools were used during the preparation of this manuscript solely for language editing and grammar checking purposes. All scientific content, data analysis, interpretation, and conclusions are the sole responsibility of the authors.

## Results

### Baseline characteristics

A total of 32 patients were included, with 16 in each group. Baseline demographic and clinical characteristics were comparable between groups (all *p* > 0.05) and are summarized in Table 1. Mean age was 69.25 ± 5.50 years in Group 1 and 67.62 ± 6.37 years in Group 2 (*p* = 0.447). No significant between-group differences were observed in sex distribution, symptom duration, preoperative BCVA, lens status, MH diameters, ELM and EZ defect sizes, or DHI.

**Table 1:** Baseline demographic and clinical characteristics of the study groups

	Conventional ILM Peeling (Group 1)	Temporal Inverted ILM Flap (Group 2)	*P* value
Age, mean ± SD, years	69.25 ± 5.50	67.62 ± 6.37	0.447
Sex (female/male), n (%)	9 (56.3%)/7 (43.7%)	11 (68.8%)/5 (31.2%)	0.465
Symptom duration, median [IQR], months	1.5 [1.0–2.0]	1.0 [1.0–2.0]	0.557
Preoperative BCVA, mean ± SD (logMAR)	1.24 ± 0.53	1.16 ± 0.46	0.818
Lens status, Phakic/Pseudophakic, n (%)	10 (62.5%)/6 (37.5%)	13 (81.25%)/3 (18.75%)	0.433
Axial length, mean ± SD, mm	23.13 ± 0.63	23.24 ± 0.66	0.634
Minimum FTMH diameter, mean ± SD (range), µm	514.00 ± 104.91 (402–765)	560.00 ± 120.42 (415–830)	0.259
Base FTMH diameter, mean ± SD (range), µm	1130.87 ± 308.46 (640–1725)	1114.25 ± 394.13 (511–2019)	0.895
Preoperative ELM defect size, mean ± SD (range), µm	1418.37 ± 380.14 (898–2194)	1496.00 ± 522.85 (714–2350)	0.635
Preoperative EZ defect size, mean ± SD (range), µm	1480.75 ± 563.50 (841–3187)	1502.31 ± 554.71 (778–2900)	0.985
Diameter hole index (DHI), mean ± SD	0.475 ± 0.106	0.538 ± 0.129	0.144

BCVA: best-corrected visual acuity; ELM: external limiting membrane; EZ: ellipsoid zone; FTMH: full-thickness macular hole; DHI: diameter hole index; IQR: interquartile range; SD: standard deviation

### Visual acuity outcomes

Mean BCVA improved from 1.24 to 0.71 logMAR in Group 1 and from 1.16 to 0.38 logMAR in Group 2 following surgery (*p* < 0.05 within each group). At 12 months, postoperative BCVA was 0.71 ± 0.46 logMAR in Group 1 and 0.38 ± 0.32 logMAR in Group 2, with significantly better BCVA in Group 2 (*p* = 0.039). The mean between-group difference, calculated as Group 1 minus Group 2, was 0.325 logMAR (95% CI, 0.05–0.59), indicating lower postoperative logMAR values in Group 2. Postoperative outcomes are presented in Table 2.

**Table 2:** Postoperative visual acuity and macular hole closure outcomes at 12 months

	Conventional ILM Peeling (Group 1)	Temporal Inverted ILM Flap (Group 2)	*P* value
Postoperative BCVA, mean ± SD (logMAR)	0.71 ± 0.46	0.38 ± 0.32	0.039*
Primary MH closure rate, n (%)	12/16 (75.0%)	16/16 (100%)	0.101
U-shaped closure, n (%)	11/12 (91.7%)	14/16 (87.5%)	0.999
V-shaped closure, n (%)	0/12 (–)	0/16 (–)	–
W-shaped closure, n (%)	1/12 (8.3%)	2/16 (12.5%)	–

BCVA: best-corrected visual acuity; MH: macular hole. *Statistically significant (*p* < 0.05)

### Macular hole closure rates and closure configuration

The primary MH closure rate was 75.0% (12/16) in Group 1 and 100% (16/16) in Group 2; however, the overall between-group difference was not statistically significant (Fisher’s exact test, *p* = 0.101). Because Group 2 had no closure failures, the uncorrected odds ratio was not finite. After application of the Haldane–Anscombe correction, the corrected odds ratio for primary closure in Group 2 versus Group 1 was 11.9 (95% CI, 0.58–241.7). The wide confidence interval, which included the null value of 1.0, reflects the substantial imprecision associated with the limited sample size and is consistent with the non-significant overall comparison. Among eyes with successful primary closure, U-shaped closure was the most common configuration in both groups, occurring in 11 of 12 eyes (91.7%) in Group 1 and 14 of 16 eyes (87.5%) in Group 2. No V-shaped closures were observed, whereas W-shaped closure was identified in 1 of 12 closed eyes (8.3%) in Group 1 and 2 of 16 closed eyes (12.5%) in Group 2. Of the 4 eyes with primary non-closure in Group 1, 2 patients declined reoperation and remained under observation, while 2 underwent repeat pars plana vitrectomy with extended ILM peeling within the 12-month follow-up period; neither achieved closure. No eye in Group 1 achieved secondary closure, and the primary closure rate therefore remained 12/16 (75.0%) at all points of assessment. Group 2 required no reintervention. An exploratory subgroup analysis stratified by minimum hole diameter showed that, among eyes with a minimum MH diameter ≥ 550 μm, primary closure occurred in 2 of 6 eyes (33.3%) in Group 1 and 8 of 8 eyes (100%) in Group 2 (Fisher’s exact test, *p* = 0.015). Both groups achieved 100% primary closure in holes measuring 400–549 μm. However, the ≥ 550 μm comparison was based on very small cell count. The cutoff was identified post hoc rather than prespecified. This subgroup result should therefore be considered exploratory and hypothesis-generating. Subgroup data are presented in Table 3.

**Table 3:** Subgroup analysis of primary macular hole closure rate by minimum hole diameter

Minimum MH Diameter	*n* (Group 1 / Group 2)	Closure Rate Group 1, *n* (%)	Closure Rate Group 2, *n* (%)	*P* value
400–549 μm	10 / 8	10/10 (100.0%)	8/8 (100.0%)	1.000
≥ 550 μm	6 / 8	2/6 (33.3%)	**8/8 (100.0%)**	0.015*
**Total (> 400 μm)**	**16 / 16**	12/16 (75.0%)	16/16 (100.0%)	0.101

MH: macular hole. P values calculated using Fisher’s exact test. *Statistically significant (*p* < 0.05)

### Outer retinal layer recovery

**Fig. 1:**
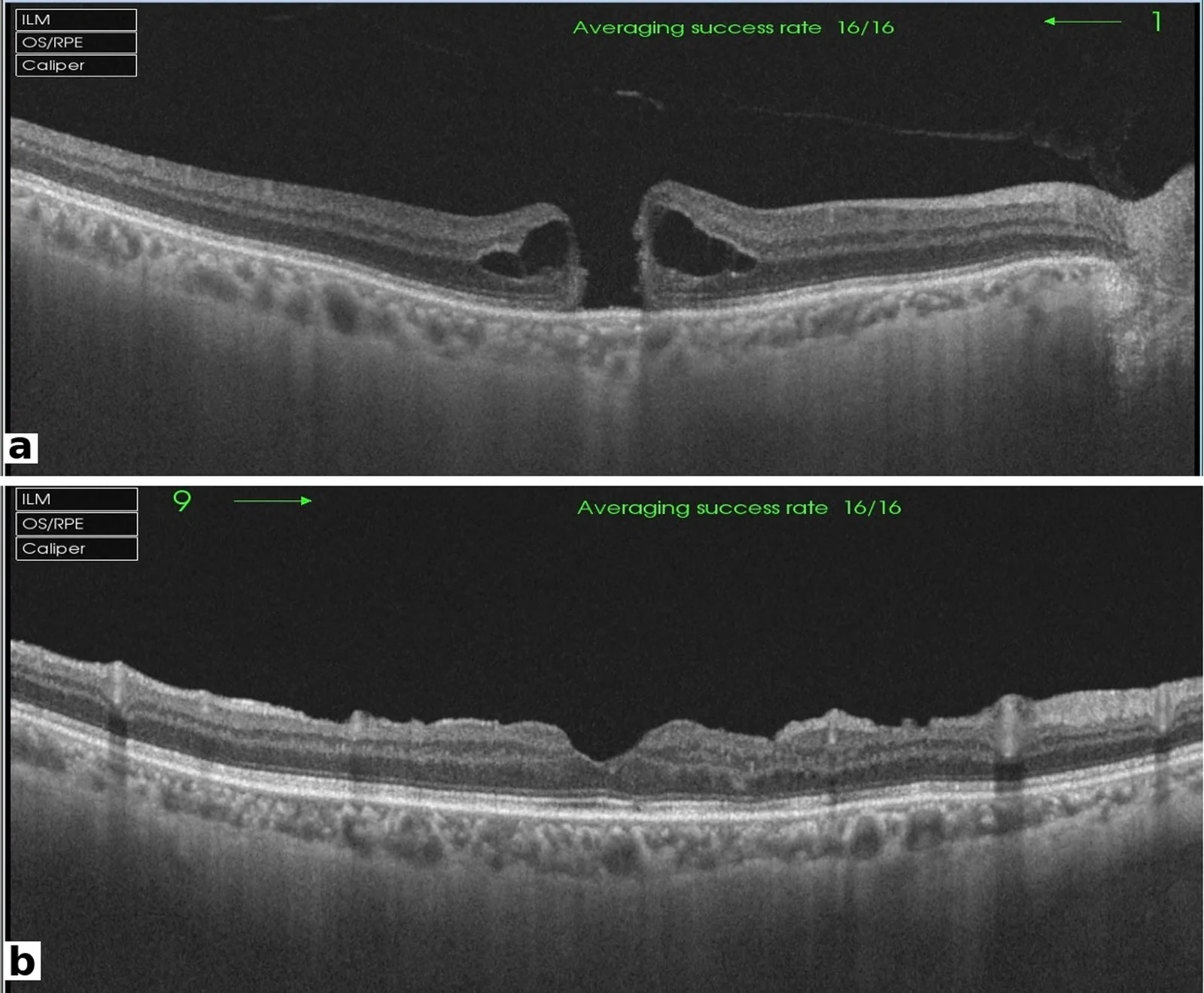
SD-OCT images of a representative patient treated with the temporal inverted ILM flap technique. Representative spectral-domain optical coherence tomography (SD-OCT) images from a patient in the temporal inverted ILM flap group (Group 2). (**A**) Preoperative SD-OCT image demonstrating a large full-thickness macular hole (FTMH) with significant disruption of the ellipsoid zone (EZ) and external limiting membrane (ELM). (**B**) Postoperative SD-OCT image at 12 months showing complete U-shaped anatomical closure of the macular hole with full restoration of both ELM and EZ integrity, consistent with favorable outer retinal layer recovery

**Fig. 2:**
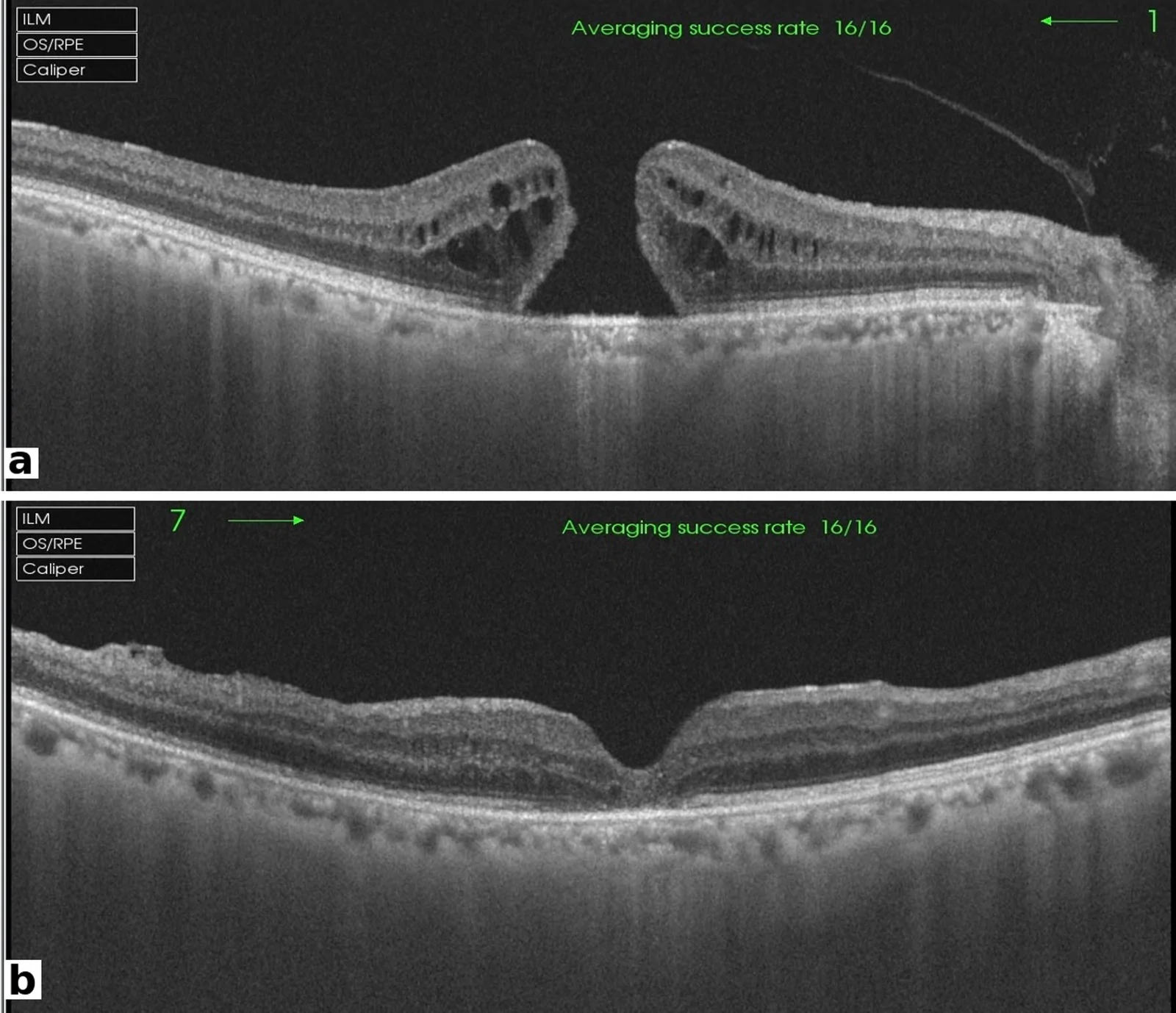
SD-OCT images of a representative patient treated with the conventional ILM peeling technique. Representative spectral-domain optical coherence tomography (SD-OCT) images from a patient in the conventional ILM peeling group (Group 1). (**A**) Preoperative SD-OCT image showing a large full-thickness macular hole with disruption of the EZ and ELM. (**B**) Postoperative SD-OCT image at 12 months demonstrating anatomical hole closure; however, persistent defects of both ELM and EZ are evident, consistent with incomplete outer retinal layer recovery despite successful anatomical closure

Mean preoperative ELM and EZ defect sizes were significantly reduced postoperatively in both groups. The mean reduction in ELM defect size was 645.8 μm in Group 1 and 1224.1 μm in Group 2, whereas the corresponding EZ defect reductions were 452.1 μm and 963.2 μm, respectively. Postoperative EZ defect size was 1028.62 ± 606.93 μm in Group 1 and 539.12 ± 618.09 μm in Group 2, with a significantly smaller defect size in Group 2 (*p* = 0.040). Postoperative ELM defect size was 772.56 ± 732.74 μm in Group 1 and 271.87 ± 495.84 μm in Group 2; this difference did not reach statistical significance (*p* = 0.056). Complete ELM restoration was observed in 37.5% of Group 1 eyes and 62.5% of Group 2 eyes (*p* = 0.157). Complete EZ restoration was observed in 6.3% of Group 1 eyes and 18.8% of Group 2 eyes (*p* = 0.600). Outer retinal layer parameters are summarized in Table 4. Representative SD-OCT images are shown in Figs. 1 and 2.

**Table 4:** Postoperative outer retinal layer parameters at 12 months

	Conventional ILM Peeling (Group 1)	Temporal Inverted ILM Flap (Group 2)	*P* value
Postoperative ELM defect size, mean ± SD, µm	772.56 ± 732.74	271.87 ± 495.84	0.056
Postoperative EZ defect size, mean ± SD, µm	**1028.62 ± 606.93**	**539.12 ± 618.09**	0.040*
Complete ELM restoration, n (%)	6/16 (37.5%)	10/16 (62.5%)	0.157
Complete EZ restoration, n (%)	1/16 (6.3%)	3/16 (18.8%)	0.600

ELM: external limiting membrane; EZ: ellipsoid zone; SD: standard deviation. *Statistically significant (*p* < 0.05)

### Exploratory multivariable analysis

In an exploratory multivariable linear regression analysis adjusting for age, lens status, DHI, baseline BCVA, and symptom duration, surgical technique remained associated with postoperative BCVA (unstandardized B = − 0.297 logMAR, 95% CI − 0.566 to − 0.027, *p* = 0.032). Baseline BCVA was also associated with postoperative BCVA (B = 0.392, 95% CI 0.095 to 0.689, *p* = 0.012), whereas age, lens status, DHI, and symptom duration were not statistically significant. Given the limited sample size relative to the number of covariates, these findings should be interpreted as exploratory (Table 5).

**Table 5:** Exploratory multivariable linear regression for postoperative BCVA (logMAR)

Predictor	Unstandardized B	95% CI	*p*
Surgical technique (flap vs. peeling)	−0.297	−0.566 to − 0.027	0.032
Age	0.007	−0.017 to 0.031	0.561
Lens status (pseudophakic)	−0.165	−0.481 to 0.152	0.293
DHI	−0.060	−1.427 to 1.307	0.929
Baseline BCVA	0.392	0.095 to 0.689	0.012
Symptom duration	0.078	−0.128 to 0.284	0.444

Model R² = 0.453; adjusted R² = 0.322; *n* = 32. A negative B for surgical technique indicates lower (better) postoperative logMAR in the flap group (Group 2). BCVA: best-corrected visual acuity; CI: confidence interval; DHI: diameter hole index; logMAR: logarithm of the minimum angle of resolution

## Discussion

In this cohort, the temporal inverted ILM flap technique was associated with better BCVA at 12 months and a smaller postoperative EZ defect size, while the overall primary closure rate did not differ significantly between the groups. A higher closure rate in the flap group was observed only in the exploratory ≥ 550 μm subgroup, which should be interpreted with caution given the small number of eyes.

An exploratory, post hoc subgroup analysis stratified by hole size suggested that the between-group difference in primary closure was concentrated among the largest holes. In eyes with a minimum MH diameter ≥ 550 μm, primary closure occurred in 2 of 6 eyes (33.3%) in Group 1 versus 8 of 8 eyes (100%) in Group 2 (*p* = 0.015), whereas both techniques achieved 100% closure in holes of 400–549 μm. This observation should, however, be interpreted with considerable caution, as the comparison rests on very small cell counts. Moreover, the ≥ 550 μm cutoff was identified post hoc rather than prespecified, and no formal threshold-optimization analysis was performed to validate it. This exploratory observation is nonetheless consistent with larger studies; Jin et al. [15], in a registry-based analysis of 2,990 eyes, reported that closure rates declined markedly in holes > 550 μm with conventional ILM peeling, while ILM flap techniques maintained high rates across all hole sizes (OR = 2.97, *p* = 0.04). Consistent with this, Steel et al. [16], in a large national cohort, identified approximately 500 μm as the point beyond which the probability of primary closure began to decline.

The higher closure rate observed with the temporal inverted ILM flap technique in the largest holes may be attributed to biological and mechanical mechanisms. The inverted ILM flap acts as a physical scaffold bridging the hole edges and supporting tissue ingrowth. Furthermore, the ILM harbors growth factors and extracellular matrix proteins, including fibronectin, laminin, and collagen IV which stimulate Müller cell proliferation and photoreceptor regeneration [17]. By repositioning the flap over the hole, these trophic factors are concentrated at the injury site, facilitating more complete anatomical and functional recovery [17, 18].

Evidence from recent meta-analyses suggests an anatomical advantage of ILM flap techniques over conventional ILM peeling. Tzoumas et al. [19] reported a higher likelihood of primary closure (OR = 4.80, *p* < 0.001), particularly in holes ≥ 500 μm. Akhtar et al. [20] also reported higher closure rates across the included hole sizes (OR = 2.47, *p* < 0.001). Similarly, the systematic review and meta-analysis by Chen et al. [21] and Reynaldo et al. [22] reported favorable anatomical outcomes with the inverted ILM flap technique compared with conventional ILM peeling. A Cochrane systematic review by Ghoraba et al. [23] concluded that the inverted ILM flap technique may increase the likelihood of macular hole closure; however, the certainty of this evidence was rated as moderate. With respect to functional outcomes, Tzoumas et al. [19] reported better visual acuity in the ILM flap group at 3–6 months (MD = − 0.14 logMAR, *p* < 0.001), which is broadly consistent with the direction of our findings.

The DHI, calculated as the minimum hole diameter divided by the base diameter, was 0.475 ± 0.106 in Group 1 and 0.538 ± 0.129 in Group 2, with no statistically significant between-group difference (*p* = 0.144). Although DHI has been investigated as a morphological index in macular hole surgery, its reported prognostic associations have been inconsistent. Therefore, no specific prognostic DHI threshold was inferred from the present data [24].

Postoperative EZ defect size was significantly smaller in Group 2 than in Group 1 (*p* = 0.040), whereas the between-group difference in postoperative ELM defect size was not statistically significant (*p* = 0.056). These findings are consistent with Baumann et al. [25] and Koçak et al. [26], who reported significantly higher rates of ELM and EZ restoration with ILM flap techniques. The prevalence of U-shaped closure among closed eyes was 91.7% in Group 1 and 87.5% in Group 2, which may have contributed to favorable visual outcomes, as U-shaped closure is consistently associated with superior foveal architecture [12, 14, 26, 27].

W-shaped closure, observed in 8.3% of Group 1 and 12.5% of Group 2 eyes, is a less frequently reported configuration associated with residual photoreceptor disruption and intermediate visual outcomes [12, 27]. Its prognostic significance remains less defined in the current literature.

Several limitations of this study merit consideration. The retrospective design introduces the potential for selection bias. A post-hoc power analysis revealed a Cohen’s d of 0.810 for BCVA, yielding an achieved power of 62.9% with 16 patients per group at an alpha level of 0.05; approximately 24 patients per group would be required to achieve 80% power. This limited power may account for the lack of statistical significance in some secondary outcome measures.

All procedures were performed by a single experienced vitreoretinal surgeon, which reduced inter-surgeon variability but may limit the external validity and generalizability of the findings. The surgeon had extensive prior experience in vitreoretinal and macular hole surgery before the study period; therefore, adoption of the temporal inverted ILM flap technique represented a technique-specific modification within an established surgical practice rather than an early general surgical learning phase. Nevertheless, because surgical technique was chronologically linked to the study period, a technique-specific adaptation effect and other unmeasured temporal factors cannot be excluded. The observed differences should therefore be interpreted as associations rather than definitive evidence of a causal treatment effect. Future prospective, ideally randomized, multi-surgeon studies with larger cohorts are warranted.

Overall, our findings suggest that the temporal inverted ILM flap technique may be a favorable option for large macular holes, particularly for the largest holes (≥ 550 μm), where it was associated with a higher closure rate in an exploratory subgroup analysis. However, given the retrospective, non-randomized design and limited sample size, this observation should be regarded as hypothesis-generating and requires confirmation in prospective, adequately powered studies before firm treatment recommendations can be made. For holes of 400–549 μm, both techniques achieved complete closure in this cohort, and the better outer retinal recovery and visual outcomes associated with the flap technique in this size range likewise warrant prospective confirmation.

## Conclusions

In this retrospective, non-randomized cohort, the temporal inverted ILM flap technique was associated with better postoperative BCVA and a smaller postoperative EZ defect size than conventional ILM peeling. The overall primary closure rate did not differ significantly between the groups (*p* = 0.101), although an exploratory post hoc subgroup analysis suggested a higher closure rate with the flap technique in eyes with a minimum MH diameter ≥ 550 μm. These findings suggest a potential functional and anatomical benefit of the temporal inverted ILM flap technique in large macular holes. However, given the retrospective, single-surgeon design, limited sample size, and small subgroup counts, these findings should be regarded as hypothesis-generating rather than confirmatory. Prospective, adequately powered, ideally multi-surgeon studies with longer follow-up are needed before firm treatment recommendations can be made.

## Data Availability

The datasets used and analyzed during the current study are available from the corresponding author on reasonable request.

## Declarations

### Authors' information

SAD is a vitreoretinal surgeon and Assistant Professor at Selçuk University Faculty of Medicine, with a subspecialty interest in macular surgery. GGC is an ophthalmologist at Konya City Hospital, and ŞG is an ophthalmologist at Selçuk University Faculty of Medicine, Konya, Turkey.

## Abbreviations

BCVA: Best-corrected visual acuity
ELM: External limiting membrane
EZ: Ellipsoid zone
FTMH: Full-thickness macular hole
ILM: Internal limiting membrane
IFT: Inverted flap technique
logMAR: logarithm of the minimum angle of resolution
MH: Macular hole
DHI: Diameter hole index
OCT: Optical coherence tomography
SD-OCT: Spectral-domain optical coherence tomography
